# Risk factors for complete recovery of adults after weaning from veno-venous extracorporeal membrane oxygenation for severe acute respiratory failure: an analysis from adult patients in the Extracorporeal Life Support Organization registry

**DOI:** 10.1186/s40560-020-00480-1

**Published:** 2020-08-20

**Authors:** Hye Ju Yeo, Yun Seong Kim, Dohyung Kim, ELSO Registry Committee, Woo Hyun Cho

**Affiliations:** 1 Division of Pulmonary, Allergy, and Critical Care Medicine, Department of Internal Medicine, Pusan National University Yangsan Hospital, Geumo-ro 20, Beomeo-ri, Mulgeum-eup, Yangsan-si, Gyeongsangnam-do 626-770 Republic of Korea; 2 Department of Thoracic and Cardiovascular Surgery, Pusan National University Yangsan Hospital, Yangsan, South Korea; 3 ECMO Registry of the Extracorporeal Life Support Organization (ELSO), Ann Arbor, MI USA

**Keywords:** Extracorporeal membrane oxygenation, Survival, Recovery, Complications

## Abstract

**Background:**

As extracorporeal membrane oxygenation (ECMO) has been widely used, the patient quality of life following ECMO termination has become an important issue as same as the patient’s survival. To date, the factors affecting complete recovery of adult survivors from ECMO have not been investigated.

**Methods:**

Data from adult patients in the Extracorporeal Life Support Organization registry who received veno-venous ECMO between 2012 and 2017 were analyzed. Multivariate logistic regression analyses were conducted.

**Results:**

In total, 6536 patients with 242,183 days of veno-venous ECMO were reviewed. The overall survival to discharge rate after weaning from ECMO was 89.7% (*n* = 5861), and 10.3% (*n* = 675) of the patients died during hospitalization. The discharge location varied as follows: 33.7% (*n* = 1976) returned home, 23.4% (*n* = 1369) were transferred to a referral hospital, 41.8% (*n* = 2447) required hospital services, and 0.6% (*n* = 36) were discharged to other places. The patients were divided into two groups according to the discharge location: a complete recovery group (*n* = 1976) and a partial recovery group (*n* = 3885). In the multivariate analyses, age (≥ 65 years) (odds ratio (OR) 0.72, 95% confidence interval (CI) 0.59–0.87, *p* = 0.001), cardiac arrest before ECMO (OR 0.76, 95% CI 0.60–0.96, *p* = 0.021), vasopressor use (OR 0.73, 95% CI 0.64–0.83, *p* < 0.001), renal replacement therapy (OR 0.40, 95% CI 0.28–0.57, *p* < 0.001), ECMO-related complications (OR 0.76, 95% CI 0.67–0.85, *p* < 0.001), and long-term ECMO support (≥ 2 weeks) (OR 0.42, 95% CI 0.37–0.48, *p* < 0.001) were significantly associated with complete recovery.

**Conclusion:**

Complete recovery after veno-venous ECMO support is associated with the patient’s baseline condition, ECMO duration, and ECMO-related complications. Respiratory ECMO should aim to increase both the survival and the quality of life after weaning from ECMO.

## Introduction

Survivors of acute respiratory distress syndrome (ARDS) often experience mental, physical, social, and functional impairments following hospital discharge [1–3]. In previous studies, a substantial discrepancy between survival to discharge and long-term survival after ARDS was observed, and late mortality was increased by age and comorbidities, not the initial severity of the ARDS [4, 5]. Since then, many studies have focused on the quality of life of ARDS survivors [6–9].

ECMO is recommended for the most severe form of ARDS as a lifesaving treatment. It is clear that this subset of patients will experience the sequelae of ARDS and critical illness, but data is lacking. In a previous multicenter cohort study of respiratory ECMO, 25.7% of the patients died in hospital after weaning from ECMO, and a further 6.9% died in the first 6 months following hospital discharge [10]. A substantial proportion of patients experienced significant unexpected re-exacerbation of ARDS after weaning from ECMO and were at risk of physical, functional, and psychological complications [11–14]. Accordingly, as same as the patient’s survival, the patient’s quality of life after ECMO termination has become an important issue as its use has increased. Understanding the outcome after ECMO weaning and identifying the risk factors affecting complete recovery are crucial for the improvement of the long-term outcome of ECMO support. There have been several studies of the factors associated with mortality after weaning from ECMO, but the focus was not on complete recovery [15–17]. In this study, we evaluated the unfavorable factors for complete recovery of adults after weaning from veno-venous (VV) ECMO for severe acute respiratory failure.

## Materials and methods

**Fig. 1:**
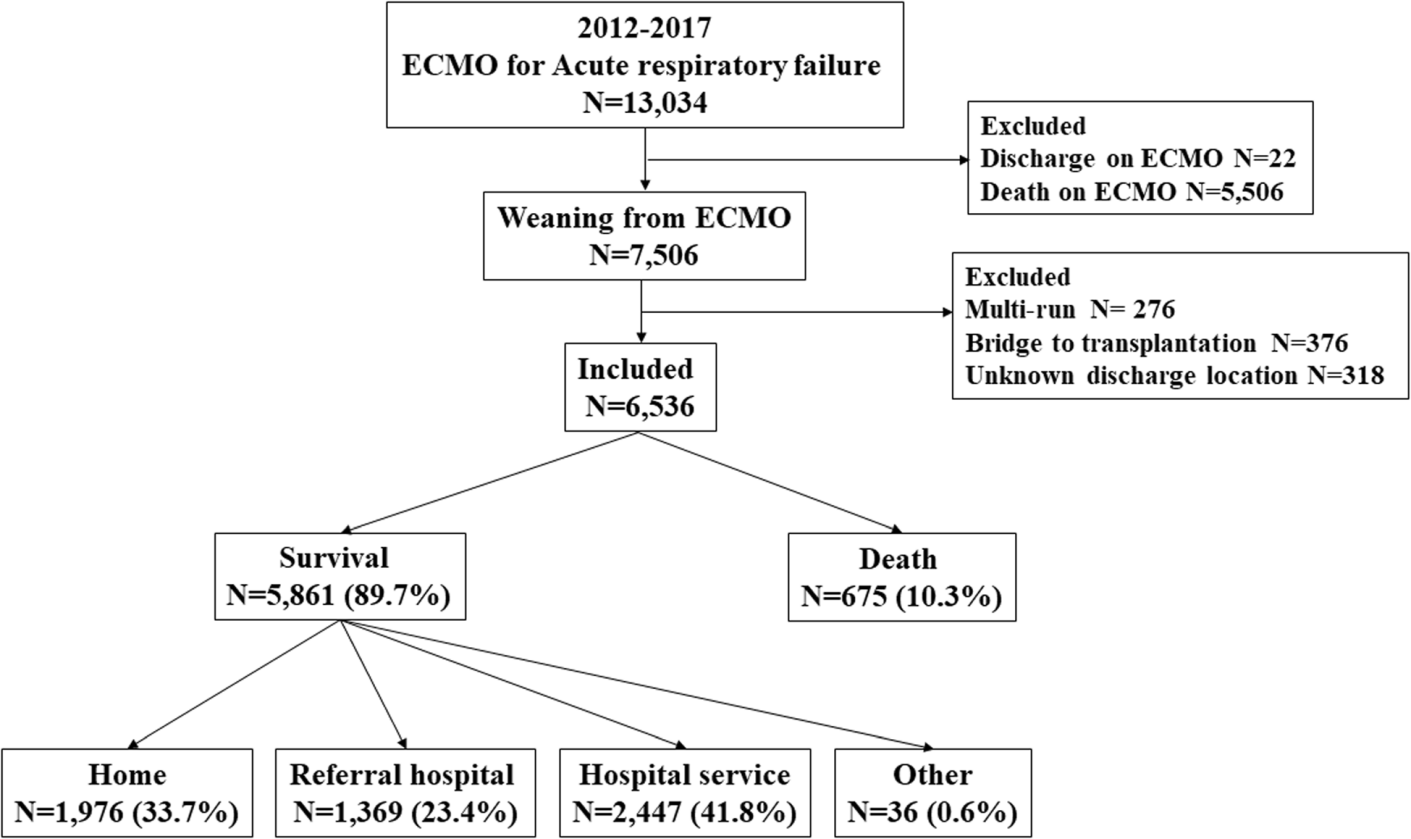
Post-weaning outcomes in veno-venous extracorporeal membrane oxygenation (ECMO)

The study design and data protection methods were presented to the Extracorporeal Life Support Organization (ELSO) steering committee, which allowed us to conduct a retrospective analysis of the ELSO registry data. This voluntary database collects baseline and outcome data on patients undergoing ECMO treatment in participating centers, with a total of 784 centers contributing until 2017. The data include age, sex, weight, primary and other diagnoses, discharge location, basic ventilation data, hemodynamic variables, arterial blood gas results, and clinical outcomes, including ECMO complications. All VV ECMO records between the years 2012 and 2017 were extracted from the ELSO database, excluding those for pediatric and neonatal patients, patients on veno-arterial ECMO (*n* = 13,034). For the analysis of the post-weaning outcomes, we excluded patients who were discharged with ECMO (*n* = 22) and those who died while receiving ECMO (*n* = 5506). The other exclusion criteria included multiple ECMO runs, where ECMO was used as a bridge to transplantation, and unknown discharge location (*n* = 970). Missing data were not imputed. Post-weaning outcomes were presented as survival to discharge or death. Survivors were classified into two groups, namely, complete recovery or partial recovery, depending on the discharge location. The complete recovery (CR) group comprised patients who were discharged to home, whereas the partial recovery (PR) group comprised patients who required hospital services, were transferred to a referral hospital, or were discharged to other places (Fig. 1). The analysis was approved by the institutional review board (Pusan National University Yangsan Hospital, 05-2019-135), and the need for informed consent was waived.

### Statistical analyses

Continuous variables were examined for normality using the Shapiro–Wilk test. Normally distributed variables were compared using the Student’s *t* test, whereas non-normally distributed variables were compared using the Kruskal–Wallis test. Categorical variables were examined using Fisher’s exact test or the chi-squared test. A value of *p* < 0.05 was defined as statistically significant. To evaluate the factors associated with CR after weaning from ECMO, logistic regression analysis was conducted. All potential clinical factors were evaluated using univariate analysis, and multivariate logistic regression analysis was conducted for variables with *p* value < 0.05. The backward stepwise method was used for multivariate analysis, with entry and removal p values set at 0.05. Statistical analysis was performed using the R software (R Foundation for Statistical Computing, Vienna, Austria, 2014).

## Results

In total, 6536 patients with 242,183 days of VV ECMO were analyzed (Fig. 1). The overall survival to discharge rate after weaning from ECMO was 89.7% (*n* = 5861), and 10.3% (*n* = 675) of the patients died during hospitalization. The discharge location varied as follows: 33.7% (*n* = 1976) returned home, 23.4% (*n* = 1369) were transferred to a referral hospital, 41.8% (*n* = 2447) required hospital services, and 0.6% (*n* = 36) were discharged to other places. The patients were divided into two groups: the CR group (*n* = 1976) and the PR group (*n* = 3885).

### Baseline characteristics and rescue therapy before ECMO initiation

Summary characteristics are presented and compared between the groups in Table 1. Significant differences in sex, age, and body weight were observed when the CR group was compared with the PR group (males 57.3% vs 60.9%, *p* = 0.009; mean age 42.3 vs 45.9 years, *p* < 0.001; mean body weight 85.9 vs 91.6 kg; *p* < 0.001). The distribution of primary diagnosis was different between two groups (*p* < 0.001). In the CR group, bacterial pneumonia and viral pneumonia were less common (bacterial 1.9% vs 3.0%, viral 18.1% vs 25.8%, *p* < 0.001). The ventilator settings and hemodynamics before ECMO initiation were significantly different between the two groups: the mean peak inspiratory pressure, PF ratio, and arterial blood pressure were higher in the CR group (*p* = 0.017, *p* = 0.005, and *p* < 0.001, respectively). Cardiac arrest before ECMO initiation, renal replacement therapy (RRT) use, and a vasopressor requirement were less common in the CR group (*p* = 0.039, *p* < 0.001, and *p* < 0.001, respectively). The use of rescue therapies before ECMO initiation significantly differed between the CR and PR groups (inhaled NO 11% vs 9.2%, *p* = 0.030; neuromuscular block (NMB) agent 28.9% vs 32.3%, *p* = 0.009).

**Table 1:** Baseline characteristics before initiation of extracorporeal membrane oxygenation (ECMO)

Variables	CR (*n* = 1976)	PR (*n* = 3885)	*p*
Male	1057 (57.3)	2336 (60.9)	0.009
Age	42.3 ± 15.2	45.9 ± 14.7	< 0.001
Weight	85.9 ± 27.1	91.6 ± 29.2	< 0.001
Primary diagnosis			< 0.001
Bacterial pneumonia	38 (1.9)	117 (3.0)	
Viral pneumonia	357 (18.1)	1004 (25.8)	
COPD/asthma	123 (6.2)	208 (5.4)	
Interstitial lung disease	174 (8.8)	122 (3.1)	
Trauma and burn	113 (5.7)	218 (5.6)	
ARDS	656 (33.2)	1145 (29.5)	
Aspiration pneumonitis	15 (0.8)	32 (0.8)	
Sepsis	29 (1.5)	69 (1.8)	
Others	471 (23.8)	970 (25.0)	
Ventilator before ECMO			
PIP	35.1 ± 10.8	34.2 ± 9.1	0.017
PEEP	13.0 ± 5.5	13.1 ± 5.7	0.674
*PaO2 FiO2 ratio*	90.8 ± 107.5	75.7 ± 188.9	0.005
Mean ABP	74.7 ± 19.2	72.2 ± 16.9	< 0.001
Cardiac arrest before ECMO	111 (5.6)	273 (7.0)	0.039
RRT	38 (1.9)	213 (5.5)	< 0.001
Vasopressor	447 (22.6)	1186 (30.5)	< 0.001
Rescue therapy before ECMO			
Systemic steroids	158 (8.0)	345 (8.9)	0.253
NO inhalation	218 (11.0)	359 (9.2)	0.030
NMB agents	572 (28.9)	1254 (32.3)	0.009

Values are expressed as mean ± standard deviation or *n* (%)

*COPD* chronic obstructive pulmonary disease, *ARDS* acute respiratory distress syndrome; *ECMO* extracorporeal membrane oxygenation, *PIP* peak inspiratory pressure, *PEEP* positive end expiratory pressure, *ABP* arterial blood pressure, *RRT* renal replacement therapy, *NO* nitric oxide, *NMB* neuromuscular blockade

### Outcomes related to ECMO and post-weaning outcomes

The clinical outcomes of ECMO are outlined in Table 2. The mean ECMO duration (days) was shorter (*p* < 0.001), and the proportion of long-term ECMO support (≥ 2 weeks) was significantly lower in the CR group (*p* < 0.001). The rate of ECMO-related complications was significantly lower in the CR group (56.9% vs 67.7%, *p* < 0.001), including cardiovascular, mechanical, neurological, pulmonary, and renal complications (Table 2).

**Table 2:** Outcomes related to extracorporeal membrane oxygenation (ECMO)

Variables	CR (*n* = 1976)	PR (*n* = 3885)	*p*
ECMO duration (days)	29.8 ± 34.6	41.1 ± 43.6	< 0.001
Long-term ECMO support (≥ 2 weeks)	1362 (68.9))	3281 (84.5)	< 0.001
ECMO complications	1125 (56.9)	2630 (67.7)	< 0.001
Cardiovascular	523 (26.5)	1350 (34.7)	< 0.001
Hemorrhage	362 (18.3)	780 (20.1)	0.108
Infection	250 (12.7)	557 (14.3)	0.077
Limb	15 (0.8)	46 (1.2)	0.130
Mechanical	364 (18.4)	907 (23.3)	< 0.001
Metabolic	291 (14.7)	633 (16.3)	0.120
Neurological	27 (1.4)	139 (3.6)	< 0.001
Pulmonary	115 (5.8)	286 (7.4)	0.027
Renal	489 (24.7)	1439 (37.0)	< 0.001

Values are expressed as mean ± standard deviation or *n* (%)

*CR* complete recovery, *PR* partial recovery, *ECMO* extracorporeal membrane oxygenation

**Fig. 2:**
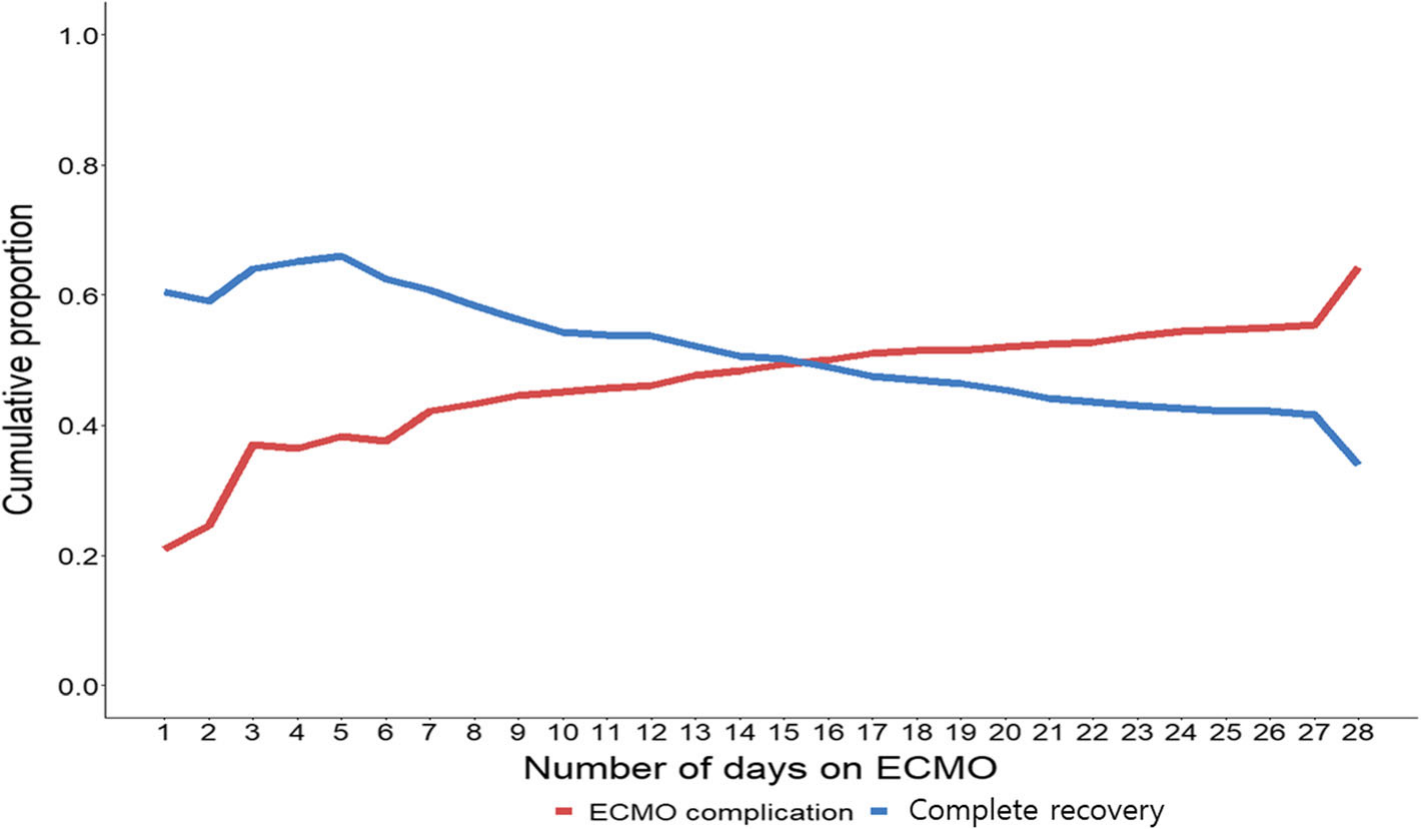
Cumulative proportion of complete recovery and extracorporeal membrane oxygenation (ECMO) complications according to the ECMO duration

The rates of CR and ECMO complications were inversely related to ECMO duration (Fig. 2). As the ECMO duration increased, the cumulative incidence of ECMO complications increased, but the cumulative proportion of CR decreased.

### Unfavorable factors for CR after weaning from ECMO

In the univariate analysis, age (≥ 65 years) (OR 0.81, 95% CI 0.68–0.98, *p* = 0.030), cardiac arrest before ECMO (OR 0.79, 95% CI 0.63–0.99, *p* = 0.040), vasopressor use (OR 0.67, 95% CI 0.59–0.75, *p* < 0.001), neuromuscular blocking agent use (OR 0.86, 95% CI 0.76–0.96, *p* = 0.009), RRT (OR 0.34, 95% CI 0.24–0.48, *p* < 0.001), ECMO-related complications (OR 0.63, 95% CI 0.56–0.71, *p* < 0.001), and long-term ECMO support (≥ 2 weeks) (OR 0.41, 95% CI 0.36–0.47, *p* < 0.001) were significantly associated with CR (Table 3).

**Table 3:** Unfavorable factors for complete recovery after weaning from veno-venous extracorporeal membrane oxygenation (VV ECMO)

Variables	Univariate regression		Multivariate regression	
	OR (95% CI)	*p*	OR (95% CI)	*p*
Age (≥ 65 years)	0.81 (0.68–0.98)	0.030	0.72 (0.59–0.87)	0.001
Cardiac arrest before ECMO	0.79 (0.63–0.99)	0.040	0.76 (0.60–0.96)	0.021
Vasopressor	0.67 (0.59–0.75)	< 0.001	0.73 (0.64–0.83)	< 0.001
NMB agents	0.86 (0.76–0.96)	0.009		
RRT before ECMO	0.34 (0.24–0.48)	< 0.001	0.40 (0.28–0.57)	< 0.001
ECMO-related complication	0.63 (0.56–0.71)	< 0.001	0.76 (0.67–0.85)	< 0.001
Long-term ECMO support (≥ 2 weeks)	0.41 (0.36–0.47)	< 0.001	0.42 (0.37–0.48)	< 0.001

*OR* odds ratio, *CI* confidence interval, *ECMO* extracorporeal membrane oxygenation, *NMB* neuromuscular blockade, *RRT* renal replacement therapy

**Fig. 3:**
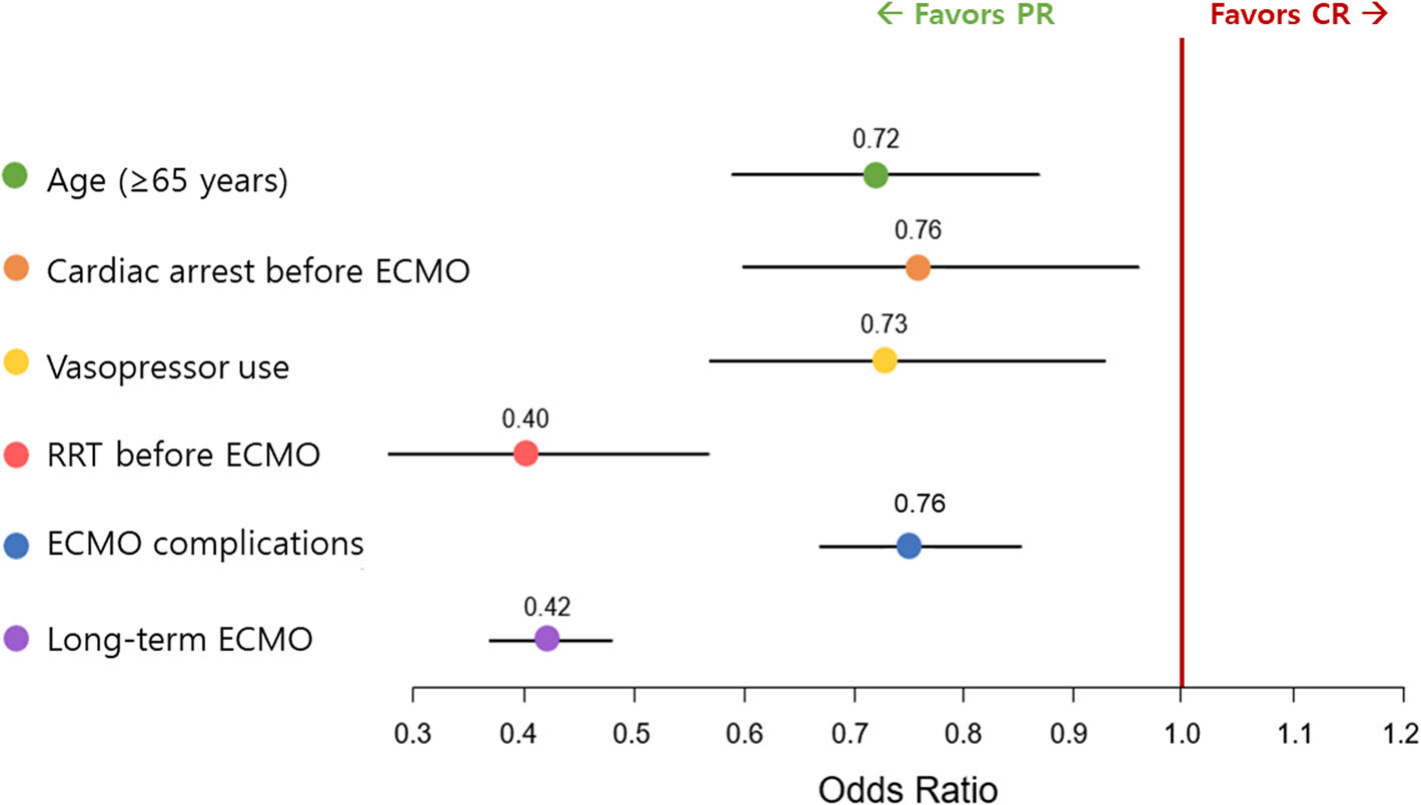
Logistic regression odds ratios of each factor comparing complete recovery with partial recovery

In the multivariate analyses, age (≥ 65 years) (OR 0.72, 95% CI 0.59–0.87, *p* = 0.001), cardiac arrest before ECMO (OR 0.76, 95% CI 0.60–0.96, *p* = 0.021), vasopressor use (OR 0.73, 95% CI 0.64–0.83, *p* < 0.001), RRT (OR 0.40, 95% CI 0.28–0.57, *p* < 0.001), ECMO-related complications (OR 0.76, 95% CI 0.67–0.85, *p* < 0.001), and long-term ECMO support (≥ 2 weeks) (OR 0.42, 95% CI 0.37–0.48, *p* < 0.001) were significantly associated with CR (Fig. 3). The odds ratio of each of the ECMO-related complications for CR is presented in Additional file 1.

## Discussion

This study revealed that only one third of patients returned home, and a substantial proportion of patients required additional hospitalization or hospital services despite surviving to discharge. The age, cardiac arrest before ECMO, severity of organ failure (vasopressor or RRT use), ECMO-related complications, and long-term ECMO support had unfavorable effects on CR. These results indicate that not only the patient’s baseline condition but also the ECMO duration and ECMO-related complications are important for CR following ECMO support, and survival does not guarantee CR.

In this study, the proportion of ECMO survivors who required transfer to another hospital or continued hospital services was higher than in other critical care populations [18–20]. The prolonged use of medical services in many survivors implied that a substantial proportion of survivors did not achieve a CR when compared with survivors of other critical illnesses. The long-term prognosis of ARDS in the pre-ECMO era is mainly affected by non-modifiable factors, such as age and initial comorbidities [20–22]. These are different from those in a specific population of respiratory ECMO patients. Recovery status was associated with ECMO-related factors in addition to baseline characteristics, such as age and initial severity. In clinical practice, long-term maintenance of ECMO means that the patient’s lung recovery is slow, which could be related to the underlying pathology requiring ECMO. However, the extended use of ECMO could inevitably be followed by several ECMO complications, as we and others have found (Fig. 2) [23, 24].

ECMO complications have a significant adverse impact on the long-term prognosis of survivors, failure to wean from ECMO, and early mortality [25–27]. This is a noteworthy point in that ECMO-related complications are potentially modifiable and could be improved. Our findings could be the basis of further improvements in ECMO care by focusing on the reduction of cardiovascular, neurological, and renal complications (Supplement 1).

This study has several limitations. First, this registry relies on voluntary reporting and may have a selection bias with such a heterogeneous group of patients. There are concerns that cases with poor clinical outcomes may be underreported, but this data reflect the clinical course after respiratory ECMO. Secondly, the functional status of the patient after discharge was only estimated on the basis of the discharge locations, and the health-related quality of life or respiratory function of the survivors was not evaluated. Due to limited information from the original registry, the possibility of overestimates of CR exists. Third, this registry did not include information regarding specific treatments for the primary diagnosis. Therefore, we cannot evaluate the impact of specific treatments for primary diagnosis on patient outcomes. Fourth, this registry did not include information about weaning strategy of ECMO and other adjunctive strategies of mechanical ventilation. Despite these limitations, the main strength of our study is the large scale of the cohort. The long-term maintenance of ECMO and ECMO complications clearly contribute to worse post-weaning outcomes in ECMO survivors. Therefore, clinicians should pay attention to modifiable factors to improve the rate of CR of patients after ECMO.

## Conclusions

In conclusion, on the basis of a large scale international cohort, a substantial number of patients with severe acute respiratory failure who successfully terminated ECMO did not achieve CR. The overall home discharge rate was only 33.7%, and old age, baseline organ failure, delayed resolution of respiratory dysfunction, and ECMO complications hindered recovery after weaning from ECMO. Future research should focus on the reduction of the modifiable risk factors to facilitate the CR of patients after ECMO.

## Supplementary information

**Additional file 1.** Logistic regression odds ratio for complete recovery of each ECMO complication after adjusting for age, severity, and ECMO duration.

## Data Availability

Not applicable
