# Supplementary material for: Risk factors for complete recovery of adults after weaning from veno-venous extracorporeal membrane oxygenation for severe acute respiratory failure: an analysis from adult patients in the Extracorporeal Life Support Organization registry
Source: J Intensive Care. 2020 Aug 20;8:64. doi: 10.1186/s40560-020-00480-1 (PMC7439234; doi:10.1186/s40560-020-00480-1)
Supplement: Supplementary file 1 — Additional file 1. Logistic regression odds ratio for complete recovery of each ECMO complication after adjusting for age, severity, and ECMO duration. [file 40560_2020_480_MOESM1_ESM.docx]

**Additional file 1: Logistic regression odds ratio for complete recovery of each ECMO complication after adjusting for age, severity, and ECMO duration**

| Adjusting age, severity, and ECMO duration | OR (95% CI) | P |
| --- | --- | --- |
| Cardiovascular complications | 0.84 (0.74–0.96) | 0.010 |
| Neurological complications | 0.46 (0.30–0.71) | <0.001 |
| Renal complications | 0.72 (0.63–0.83) | <0.001 |

Cardiovascular, neurological, and renal complications are unfavorable for complete recovery.

ECMO; extracorporeal membrane oxygenation, OR; odds ratio, CI; confidence interval .
